# Supplementary material for: Antibody Watch: Text mining antibody specificity from the literature
Source: PLoS Comput Biol. 2021 May 27;17(5):e1008967. doi: 10.1371/journal.pcbi.1008967 (PMC8189493; doi:10.1371/journal.pcbi.1008967)
Supplement: S1 Text — A. Overview of AEN and LCF. B. Siamese BiLSTM. C. Hyperparameter settings. Recently, models based on the attention-encoder network (AEN) and local context focus mechanism (LCF) models were reported to achieve state-of-the-art results for benchmark ABSA datasets. We also implemented these models and their variants for a comparison. AEN learns to build attention links within an input sentence and between the input sentence and the aspects to perform aspect-based sentiment analysis. LCF focuses on using attention mechanisms to relate local context to the aspects. The details of our implementation are given in A. A neural network model with a Siamese Recurrent Architecture consists of two recurrent neural network components trained to have identical parameters (thus the name “Siamese”) to read a pair of different input sentences. Then the difference between the output vectors of the two components is computed to determine if the input sentences are similar. We used either Manhattan or Euclidean distance as the last layer to measure the similarity between the input snippet pair for our task of RRID linking. B describes the details. The hyperparameter settings for the training are given in C. (PDF) [file pcbi.1008967.s001.pdf]

Supporting Information S1 Text

Antibody Watch: Text Mining Antibody Specificity from the Literature (Hsu et al.)

A. Overview of AEN and LCF

The Attentional Encoder Network (AEN) employs attention based encoders for the modeling, since recurrent neural networks are difficult to parallelize and may truncate the backpropagation through time, which brings difficulty in remembering long-term patterns. The network consists of four layers which are Embedding layer, Attentional Encoder layer, Target-specific Attention layer, and Output layer. In our implementation, we chose BERT as its embedding layer. The output from this layer is fed to the Attentional Encoder layer. The Attentional Encoder layer is a parallelizable and interactive alternative of LSTM and is applied to project the hidden states of the input embeddings. This layer consists of two submodules: the Multi-Head Attention (MHA) and the Pointwise Convolution Transformation (PCT). After we obtained the introspective context representation and the context-perceptive target representation, we then applied another MHA to obtain the target-specific context representation. For the Output layer, the final representation is computed by average pooling, concatenating them and using a fully connected layer to project the concatenated data.

A **Local Context Focus (LCF)** Mechanism for Aspect-Based Sentiment Classification is based on a view of local context word which is related to the target aspects by utilizing self-attention to capture local context features and global context features concurrently. This model also used Semantic-Relative Distance (SRD) to help locate the local context of each target aspect by evaluating the dependency between contextual words and target aspects. The network is separated into two parts. The first part is called global context. The input and process of this part is similar to BERT-SPC. The second part is called local context. The first part is word embedding whose input is the snippet. The second part is feature extraction or local context focus with the use of SRD to help locate the local context of each target aspect. The inputs of this feature extraction are both the snippet and aspect word. The output from global context will be concatenated with the output from local context which is the result from multiplying the output from BERT with the output from local context focus. We applied MHSA to interactively learn the features of the global context. For the Output layer, the result from the previous layer is pooled by extracting the hidden states on the corresponding position of the first token. Finally, a Softmax layer is applied to predict the sentiment polarity.

B. Siamese BiLSTM

The **Siamese Recurrent Architectures** were shown to be effective for the sentence similarity classification. They consist of two recurrent network components trained to have identical parameters (thus the name “Siamese”) to read a pair of different input sentences. Then the difference between the output of the two components is computed to determine if the input

sentences are similar. We implemented this architecture by using a bi-directional long-short term memory (biLSTM) as the recurrent layer to encode a snippet into a vectorized representation. For the last layer, we consider both Manhattan distance and Euclidean distance of the encoded vectors to measure the similarity between the input sentence pair.

C. Hyperparameter Settings

BERT dimension size = 768
The hidden layers in BiLSTM = 200
Input maximum length = 128
Batch size = 8
Optimizer: Adam with learning rate of 2e-5
Maximum epoch = 10
Drop out = 0.1

Please also see source codes for details.
https://github.com/SciCrunch/Antibody-Watch
