# Supplementary material for: Antibody Watch: Text mining antibody specificity from the literature
Source: PLoS Comput Biol. 2021 May 27;17(5):e1008967. doi: 10.1371/journal.pcbi.1008967 (PMC8189493; doi:10.1371/journal.pcbi.1008967)
Supplement: Attachment — Submitted filename: Responses.pdf [file pcbi.1008967.s002.pdf]

We would like to thank the editors for considering our manuscript entitled “Antibody watch: Text mining antibody specificity from the literature” for publication in *PLoS Computational Biology*. We also would like to thank the anonymous reviewers for their valuable comments and have thoroughly revised the manuscript to address these comments, which have greatly improved the manuscript. For the editors and reviewers’ reference, we have included below a list of revisions that we made to address the comments. We thank the editors and reviewers again for your time considering our manuscript and your constructive comments.

Reviewer #1:

Authors present a text mining-based tool to identify problematic antibodies by extracting statements regarding antibody specificity from the literature. The task is divided into two parts: Task 1 is to recognize antibody specificity snippets and labeling them as specific, non-specific, and neutral. The second task is to identify the RRID corresponding to the antibody from another text snippet, and combining them to generate antibody specificity triples. Neural network models are built for both tasks. An attention-on-attention model combined with SciBERT contextualized embeddings performs best for the first task, and a SciBERT-based sentence-pair model performs best for RRID linking task. The models are proposed for an antibody alerting knowledge base.

This manuscript reports a useful tool, and the knowledge base proposed would be a valuable resource for researchers working with antibodies. The manuscript is overall clearly written and well organized. The experimental work seems sound. Use of aspect-based sentiment analysis for the first task is sensible. Based on the examples given, the tasks (particularly the first) do not seem particularly complex (use of the words 'specific' and 'non-specific' seem quite predictive), but a couple of examples given in Discussion indicate that there can be some complex cases. I don't have a major problem with the manuscript, but I think it can improved in some aspects.

We thank the reviewer for the positive feedback.

- It can be made clearer whether a snippet is always a sentence, or whether it can be multiple sentences or a sentence fragment.

A snippet consists of at most three sentences including the sentence where the keywords appear and the sentences above and below it in the context. When the sentence containing the keywords is in the beginning or the end of a paragraph or a caption of a figure or table, a snippet may have less than three sentences. Please see Line 83-89 Page 3-4 and Line 256-260 Page 8.

- The data seems somewhat skewed due to their sentence selection criteria and may not be quite a representative sample. It would be interesting to see the results when applied to a randomly selected held-out dataset.

There are fewer statements reporting non-specific antibodies because more often authors intend to report valid experimental results using specific antibodies to draw their conclusions. It is thus expected that the data is skewed (Line 324-328 Page 9).

- The authors take the presence of RRID in a publication as a starting point. How much is being missed in terms of antibody specificity knowledge by making this choice? Could/should the approach be extended to cases/journals where RRIDs may not be used/enforced? Some discussion would be useful.

We agree that a large number of useful statements about antibody specificity appear in articles not specifying RRIDs of the antibodies, though RRIDs are increasingly required by publishers. It is our

plan to mine these statements with our task 1 model and manually (or semi-automatically) assign these statements to antibodies. We are currently developing a prototype pipeline to facilitate the whole process as a part of our dkNET service. We added a discussion about this point to Line 420-424 Page 13.

- Task 2 is cast as a sentence pair classification task. It is unclear how RRIDs are identified exactly once a pair is classified as positive. Does the RRID sentence/snippet in the pair always contain a single RRID? If multiple RRIDs are present in the snippet how is the selection done? Simply regular expressions? Any errors made here?

We thank the reviewer for the question and revised the manuscript to clarify the preprocessing steps. These steps ensure that there will be no more than one RRID in an RRID snippet to be paired with a specificity snippet. Given a mention of RRID in the text, we include at most three sentences in its context to create the snippet in the same way as when we prepare snippets for specificity statements. However, if the sentence before and after the sentence where the RRID mention appears contain other RRID mentions, they will not be included. If the sentence where the RRID mention appears contains other RRID mentions, the sentence will be split into sub-strings at RRID mentions and only the sub-string that contains the RRID mention will be retained. The sub-strings that contain other RRID mentions will be discarded.

With regard to the performance of regex for RRID mention identification, since the format of RRID is strictly standardized, identifying RRID mentions is highly accurate. We have been using a semi-automatic curation pipeline to monitor how authors specify RRIDs in publications. In our previous study reported in [28] we found that regex (RRID-by-RDW) achieved 0.944 F1-score The errors are due to malformed RRIDs by authors. As more tools are developed to support the use of RRIDs, we expect these errors to diminish. An example of such tools has been implemented by the journal eLife. Please see Line 276-282 page 8 for our revision.

- Brief descriptions of some models (AEN, LCF, and Siamese Recurrent Architecture with BiLSTM) can be provided.

The Attentional Encoder Network (AEN) employs attention based encoders for the modeling, since recurrent neural networks are difficult to parallelize and may truncate backpropagation through time, which brings difficulty in remembering long-term patterns. The network consists of four layers which are Embedding layer, Attentional Encoder layer, Target-specific Attention layer, and Output layer. In our implementation, we chose BERT as its embedding layer. The output from this layer is fed to the Attentional Encoder layer. The Attentional Encoder layer is a parallelizable and interactive alternative of LSTM and is applied to project the hidden states of the input embeddings. This layer consists of two submodules: the Multi-Head Attention (MHA) and the Pointwise Convolution Transformation (PCT). After we obtained the introspective context representation and the context-perceptive target representation, we then applied another MHA to obtain the target-specific context representation. For the Output layer, the final representation is computed by average pooling, concatenating them and using a fully connected layer to project the concatenated data. A brief description has been added (Line 218 Page 7).

A Local Context Focus (LCF) Mechanism for Aspect-Based Sentiment Classification is based on a view of local context word which is related to the target aspects by utilizing self-attention to capture local context features and global context features concurrently. This model also used Semantic-Relative Distance (SRD) to help locate the local context of each target aspect by evaluating the dependency between contextual words and target aspects. The network is separated into two parts. The first part is called global context. The input and process of this part is similar to BERT-SPC. The second part is called local context. The first part is word embedding whose input is the snippet. The second part is feature extraction or local context focus with the use of SRD to help locate the local context of each target aspect. The inputs of this feature extraction are both the snippet and aspect word. The output from global context will be concatenated with the output from local context which is the result from multiplying the output from BERT with the output from local context focus. We applied

MHSA to interactively learn the features of the global context. For the Output layer, the result from the previous layer is pooled by extracting the hidden states on the corresponding position of the first token. Finally, a Softmax layer is applied to predict the sentiment polarity. A brief description has been added (Line 220 Page 7).

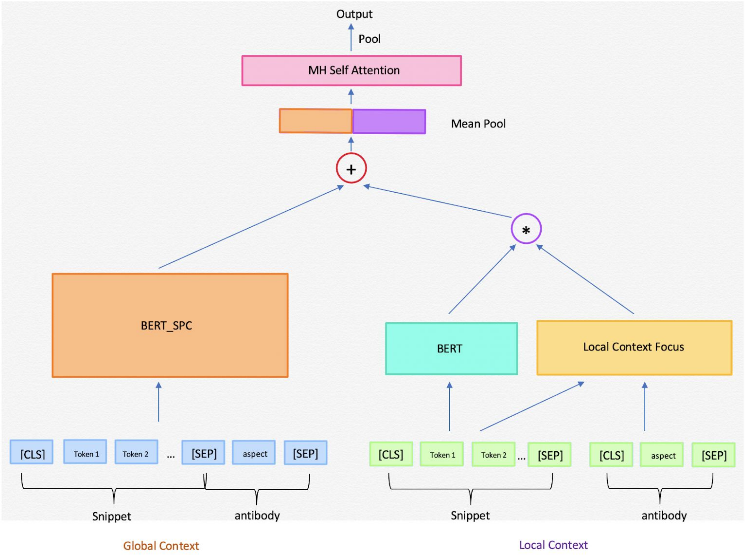

The Siamese Recurrent Architectures were shown to be effective for the sentence similarity classification. They consist of two recurrent network components trained to have identical parameters (thus the name "Siamese") to read a pair of different input sentences. Then the difference between the output of the two components is computed to determine if the input sentences are similar. We implemented this architecture by using a bi-directional long-short term memory (biLSTM) as the recurrent layer to encode a snippet into a vectorized representation. For the last layer, we consider both Manhattan distance and Euclidean distance of the encoded vectors to measure the similarity between the input sentence pair. A brief description has been added (Line 234-238 Page 8).

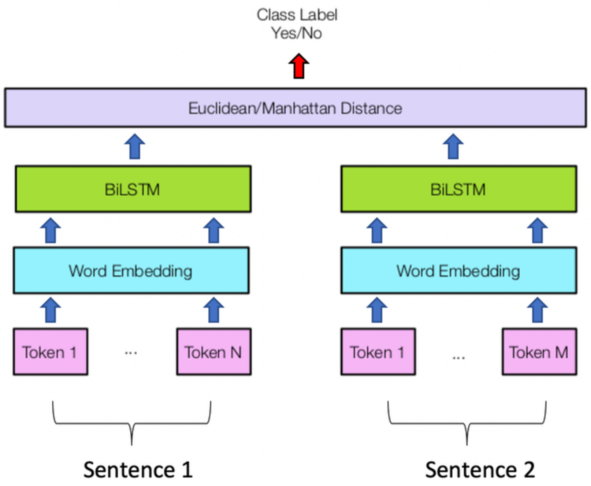

We also added the above descriptions to the Supporting Information Appendix 1.

- BioBERT seems like a more natural choice for contextualized embeddings than SciBERT. I think it could be added to this paper, rather than mentioned as future work.

We repeated all experiments for SciBERT with BioBERT and obtained the results. All results for the specificity classification task (Task 1) are given below.

Model	Positive	Negative	Neutral	Macro F1	Weighted F1
BioBERT	0.902	0.590	0.330	0.606	0.824
BioBERT-SPC	0.946	0.800	0.732	0.826	0.912
BiLSTM-AOA-BioBERT	0.932	0.750	0.690	0.790	0.885
**AOA-BioBERT**	0.956	0.796	0.746	0.830	0.918
MHA-AOA-BioBERT	0.938	0.752	0.700	0.800	0.900
AEN-BioBERT	0.936	0.796	0.676	0.782	0.888
LCF-BioBERT	0.948	0.772	0.726	0.814	0.910
CLS-AOA-BioBERT	0.940	0.732	0.714	0.794	0.898
CLS-BiLSTM-AOA-BioBERT	0.952	0.772	0.738	0.818	0.912
CLS-MHA-AOA-BioBERT	0.930	0.747	0.677	0.783	0.883
CLS-AEN-BioBERT	0.944	0.742	0.718	0.800	0.900
CLS-LCF-BioBERT	0.950	0.798	0.708	0.818	0.913

For the sake of conciseness, we only include the best BioBERT results to Table 3 and Table 4 in the manuscript. We note that while we tested BioBERT for the RRID linking task (Task 2) we also updated the numbers for BERT and SciBERT because we found that the calculation of the original scores was faulty. The updated results however do not impact the original conclusion.

- What are the hyperparameters used to train the neural networks?

The hyperparameters used to train the models are as listed:

- BERT dimension size = 768
- The hidden layers in BiLSTM = 200
- Input maximum length = 128
- Batch size = 8
- Optimizer: Adam with learning rate of 2e-5
- Maximum epoch = 10
- Drop out = 0.1

The hyperparameters for training can also be found in our Github repo: https://github.com/SciCrunch/Antibody-Watch/blob/master/ABSA/train.py for specificity classification (task 1) and https://github.com/SciCrunch/Antibody-Watch/blob/master/rrid/notebook/SciBERT.ipynb

for RRID linking (task 2). We also provide the numbers in the Supporting information Appendix 2 for readers' reference.

- A neural network architecture diagram could be useful.

The architecture diagram is given here and provided as Fig 2.

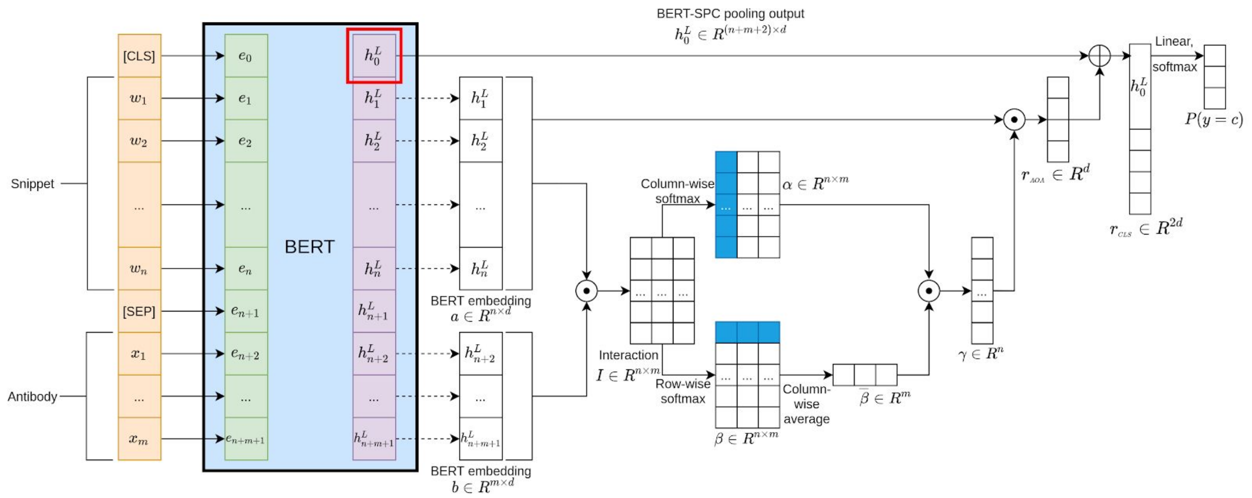

- I was surprised that the inter-rater agreement was not better than "substantial", as the tasks seem relatively straightforward. Some examples of disagreement would be useful.

The disagreement was mainly because initially "neutral" was not consistently annotated between curators, resulting in 128/720 cases being annotated as either specific or neutral. Many of them state that a specificity test for antibodies was performed. If a positive result is reported then it is specific but neutral if no result is reported. For example:

"To confirm **specificity** of each monoclonal **antibody,** an ELISA was performed…"

The above sentence is followed by a lengthy description of how the ELISA test was performed but no result was given (PMID 27342872). It should be annotated as ``neutral," though one may argue that no result given implies that no issue and thus is specific. However, we would like to train the model to classify based on given evidence instead of inferring what may be implied and decided to annotate in this way.

We added the above discussion (line 312-323 page 9).

Also in the Discussion section, we have provided remarkable examples that are challenging to both curators and the NLP models.

- It is not clear why task 2 involves 1100 sentences, when for Task 1 2639 sentences were labeled.

Table 2 shows that 2639 is the number for Task 1, 7245 for Task 2, and 1100 is the number among 7245 cases where a RRID mention snippet and a specificity snippet are linked (thus Yes). The last row of Table 2 then gives the number of each specificity class of the specificity snippets annotated in these 1,100 positive RRID linking examples. The total number of pairs for Task 2 is 7245. Please see line 324-334, page 9-10, and Table 2.

- Are antibodies always single tokens?

As an aspect term, yes. We clarified this in the caption of Fig 2 and line 347 page 10.

In a snippet, an antibody may be named in a wide variety of ways with a different number of tokens. Please see line 134-141, page 5 for a discussion.

- RRID discussion on page 12 seems out of context and can probably be cut.

Since we link specificity statements to RRIDs and only process papers containing RRID mentions, it is important to discuss how we extract from papers without RRIDs and the future provision of RRIDs. We revised the discussion to clarify our point. (RRID section now in page 13)

- Why does b = (h^L_n+2) start with n+2 instead of n+1?

Because h^L_n+1 is the output for the separator token “[SEP].” Please refer to the neural network diagram above (or Fig 2 in the manuscript) for an illustration.

- "classify such snippet into "- > snippets

Done.

- "models of baselines" -> baseline models?

Done.

- Give full name of BiLSTM when first introduced.

Done.

- concate -> concatenate

Done.

- "Note that a snippet contains three sentences": Is it "the snippet"?

Done.

- "is not always bind to" -> "does not always bind to"

Done.

- Figure 1 seems blurry, but should be fixed if accepted (Specifically the labels, e.g. PMC number, are unreadable).

A new image was created to improve the resolution.

Reviewer #2:

1. Because there is no benchmark dataset for this work, the authors developed a dataset for evaluation. However their dataset only include text containing the regex patterns of “(S|s)pecific, ((B|b)ackground staining)” or “(C|c)ross(|-)reactiv?”. Is it likely to lose some antibody specificity text, which cannot be identified by the patterns? The authors should clarify this point. What is the number of final papers in the dataset?

We thank the reviewer for the question. These regex patterns serve as a minimum filter to remove irrelevant snippets which may easily overwhelm the model. Other terms can be easily included in the data preparation steps if they are found to be useful. (line 252-255 page 8)

Meanwhile, we are working on a pipeline allowing users-in-the-loop to upload cases that they find as reporting antibody specificity issues and will include any additional terms useful to our filter.

The final dataset of snippets were from 995 papers. The number is added to line 261 page 8 in the manuscript.

2. Each snippet contains both the previous and next sentences of the main sentence. Did the authors evaluate the effects of different numbers of surrounding sentences on performances?

We thank the reviewer for the question. We agree that more or less may potentially impact the performance and may deserve investigation. The choice of three sentences at most can be considered as an assumption similar to the Markov assumption where the probability of a new state depends only on the previous state. Here our assumption is that the immediate adjacent sentences before and after the main sentences contain sufficient information while not bringing in too much irrelevant information that creates unwanted noise to burden the model. The assumption allows us to concentrate on training the model to make a classification based on three sentences in the context at a time instead of having to read the entire article. We added this discussion to line 83-89 page 3-4.

3. How did the authors divide the dataset into 5-fold? Is it possible that a pair appearing in the test fold also in the training fold?

We used the library function Kfold from Scikit-learn to perform the 5-fold cross validation. We set the parameter “shuffle=True.” The function splits the input pairs into k=5 consecutive folds with shuffling. Each fold is then used once as a validation while the k-1=4 remaining folds form the training set. A pair appearing in the test fold will never be in the training fold.

4. It seems that their baselines for the RRID-linking can also apply to the Specificity classification. Is it possible to see the baselines on the specificity classification?

Yes. BioBERT-SPC and SciBERT-SPC were tested for the specificity classification and the results are indeed competitive although slightly worse than when the aspect is considered with a more sophisticated model design. Please see Table 3 for the results.

Minor:

1. Table 1, please add the PMIDs of examples.

Done.

2. Page 6, why do you mention "(RRID:SCR 018008)" at the 191st line? Also, Page 8, "RRID:SCR 017679" at the 252nd line.

These show how to cite a research resource with its RRID, which covers not only antibodies but also digital research resources including software tools and datasets.

3. Page 6, at the 181st line, the superscript "2" of "ABSA2" is part of your system name but looks like a footnote.

We have revised all mentions of the system name by using LaTeX “\textsuperscript” command and added parenthese to make it “(ABSA)2” that we hope to reduce confusion.

4. The text of Fig 1 is unclear.

We have created a new Fig 1 to make it clear.
